# Widespread co-endemicity of *Trypanosoma* species infecting cattle in the Sudano-Sahelian and Guinea Savannah zones of Cameroon

**DOI:** 10.1186/s12917-019-2111-6

**Published:** 2019-10-16

**Authors:** Archile Paguem, Babette Abanda, Dieudonné Ndjonka, Judith Sophie Weber, Sen Claudine Henriette Ngomtcho, Kingsley Tanyi Manchang, Mamoudou Adoulmoumini, Albert Eisenbarth, Alfons Renz, Sørge Kelm, Mbunkah Daniel Achukwi

**Affiliations:** 1 Faculty of Science, University of Ngaoundéré, P.O. Box 454, Ngaoundéré, Cameroon; 2 Institute for Evolution and Ecology, Department of Comparative Zoology, University of Tübingen, Auf der Morgenstelle 28, 72076 Tübingen, Germany; 3 Centre for Biomolecular Interactions Bremen (CBIB), Faculty of Biology and Chemistry, University of Bremen, 28334 Bremen, Germany; 4 Ministry of public health, Yaoundé, Cameroon; 5 Institute of Agricultural Research for Development (IRAD), Wakwa, P.O. Box 65, Ngaoundéré, Cameroon; 6 XSchool of Veterinary Medicine and Sciences, Department of Parasitology, University of Ngaoundéré, P.O. Box 454, Ngaoundéré, Cameroon; 7 Institute of Novel and Emerging Infectious Diseases, Friedrich Löffler Institut, Federal Research Institute for Animal Health, Südufer 10, 17493 Greifswald, Insel Riems Germany; 8 TOZARD Research Laboratory, P.O. Box 59, Bambili-Tubah, Bamenda, Cameroon

**Keywords:** Trypanosomosis, ITS-1, gGAPDH, *T. grayi*, *T. Theileri*, Co-endemic trypanosomes, Cameroon

## Abstract

**Background:**

African animal trypanosomosis remains the major constraint of livestock production and livelihood of pastoral communities in Cameroon. Despite several decades of vector and parasite control efforts, it has not been eradicated. Alternative and sustainable control strategies require a sound knowledge of the local species, strains and vectors. In the Sudano-Sahelian and Guinea Savannah of Cameroon the prevalence and genetic diversity of trypanosomes infecting cattle was investigated by microscopy of cattle blood buffy coat and molecular methods using generic primers targeting parts of the internal transcribed spacer 1 (ITS-1) and encoded glycosomal glyceraldehyde 3-phosphate dehydrogenase-gene (gGAPDH).

**Results:**

A total of 1176 randomly chosen cattle from five divisions in the Sudano-Sahelian and Guinea Savannah of Cameroon were examined. The overall prevalence of trypanosomes by microscopy was 5.9% (56/953) in contrast to 53.2% (626/1176) when molecular tools were used. This indicated a limited sensitivity of microscopy in subclinical infections with frequently low parasitemia. Three trypanosome species were identified by light microscopy: *T. vivax* (2.3%), *T. brucei* (3.7%) and *T. congolense* (3.0%), whereas five were identified by PCR, namely *T. grayi/T. theileri* (30.8%), *T. vivax* (17.7%), *T. brucei* (14.5%) and *T. congolense* (5.1%). Unexpected cases of *T. grayi* (*n* = 4) and *T. theileri* (*n* = 26) were confirmed by sequencing. Phylogenetic analysis of the gGAPDH revealed the presence of *T. vivax,* clade A and *T. vivax* clade C, which were co-endemic in the Faro et Deo division.

*T. grayi/T. theileri* were the predominant species infecting cattle in tsetse free areas. In contrast, *T. vivax*, *T. brucei* and *T. congolense* were more abundant in areas where the *Glossina*-vectors were present.

**Conclusions:**

The abundance of pathogenic trypanosomes in tsetse infested areas is alarming and even more, the occurrence of *T. vivax*, *T. brucei*, *T. congolense*, *T. theileri* and *T. grayi* in tsetse-free areas implies that tsetse control alone is not sufficient to control trypanosomosis in livestock. To implement control measures that reduce the risk of spread in tsetse free areas, close monitoring using molecular tools and a thorough search for alternative vectors of trypanosomes is recommended.

## Background

In tropical Africa and South America, hemoparasitic flagellates of the genus *Trypanosoma* cause severe to fatal diseases in wild and domestic mammals, including the human host. Trypanosomes infecting mammals are divided into two major families: *Salivaria* and *Stercoraria* [1]. Members of the *Salivaria* include human and veterinary medically important pathogens *Trypanosoma vivax, T. congolense* and *T. brucei* spp. They develop as mammalian infective forms in the mouthparts, e.g. proboscis and salivary glands of tsetse (*Glossina* spp.). Transmission to the vertebrate host occurs during the blood meal of an infective tsetse [2]. In contrast, the *Stercoraria* comprise the South American parasite *T. cruzi* and the worldwide-distributed *Megatrypanum,* e.g. *T. theileri,* where the final stages of the parasite develop in the posterior digestive tract of the arthropod vectors. These species are transmitted by contamination of the bite puncture with infectious excreta from the vector [3]. Trypanosomes can also be transmitted by mechanical vectors, like tabanid and stomoxine horse flies and by hard-ticks [4, 5].

In Cameroon, 90% of the population of the estimated six million cattle are at risk of trypanosome infection [6]. The Adamawa highland plateau in North Cameroon is the country’s main area of cattle rearing supplying animal products to all neighboring countries. This was made possible through the control of tsetse on this up to 1000 m high plateau [7], whilst *Glossina morsitans*, *G. fuscipes fuscipes* and *G. tachinoides* still occur in high numbers in the savannah pastures of the Eastern and Northern regions making cattle rearing problematic [7]. However, conventional operations employed over the last three decades have not eradicated the *Glossina* vectors so that pasture lands previously cleared and declared free of *Glossina* have recently been re-invaded [7, 8]. Disease control in these areas depended on continuing diagnosis and treatment of suspected cases with the few trypanocidal drugs available on the market [9]. Isometamidium, diminazene and homidium bromide are the only drugs widely used during more than four decades for trypanosome control. Furthermore, there are reports of drug resistance coming from North Cameroon [10] and elsewhere [11, 12]. Therefore, the unequivocal identification of the prevailing trypanosome species and strains has gotten more attention to prevent unnecessary treatment of non-pathogenic parasites and thereby promoting the development of resistance.

In Northern Cameroon, little is yet known about the genetic diversity of trypanosomes infecting cattle. Most epizootiological data available were based on microscopy, such as phase-contrast or dark field examination of the buffy coat, thin or thick blood smears, and to a lesser extent also serological analyses [13–15]. These investigations indicated *T. congolense*, *T. brucei* and *T. vivax* as the only prevalent species in these areas [7–10]. Microscopy, albeit easy to perform in a fieldwork setting, needs a high investment in time and training, risks to misinterpret rare, emerging or in other ways unexpected specimens and fails to detect immature infections during the first stages of infection [16]. Advances in molecular biology have expanded the limits of the traditional methods in sensitivity and specificity. Generic and specific primers have been designed to amplify the internal transcribed spacer 1 (ITS-1) region of the ribosomal RNA gene locus of trypanosomes, chosen because of its high copy number and inter-species length variation [17–19]. Thus, trypanosome species are recognizable by the fragment length of their PCR-amplified ITS-1 region [17]. This method has evolved to improve sensitivity and detection of trypanosomes in animal blood [18–20]. In addition, the glycosomal glyceraldehyde 3-phosphate dehydrogenase gene (gGAPDH), an ubiquitous and essential glycolytic enzyme, has been used for the species differentiation of trypanosomes because of its lower rate of molecular evolution [21]. Despite the fact that it has no band size separation among different *Trypanosoma* species, it has been a marker of choice for phylogenetic analysis [22, 23].

A recent study in two restricted areas in Northern Cameroon relying on molecular tools for parasite detection [24] revealed active foci of AAT on the Adamawa region in the Faro et Deo close to the border with Nigeria and in the North region near the town of Gamba. The results revealed the crucial need of molecular tools to monitor the diversity of trypanosomes together with their vectors in hyper-endemic foci. A higher diversity of trypanosomes was seen in cattle and tsetse vectors than previously known. Those observations were however based only on a few *Glossina*-infested localities and on less than four hundred cattle examined. Therefore, this study has investigated the epizootiological picture of bovine trypanosomosis in the northern regions of Cameroon comparing tsetse infested areas in the high Guinea savannah and the Sudano-Sahelian zone with areas cleared of tsetse in both agro-ecological zones. Furthermore, the different susceptibilities of the various indigenous cattle breeds found in these zones have been addressed.

Gudali, White Fulani and Red Fulani are the major local zebu cattle breeds [25]. They are claimed to be more susceptible to trypanosomiasis than the autochthonous taurine cattle breed called Namchi (Doayo) [26], which nevertheless is at high risk of becoming extinct [27]. Only few located herds of Doayo cattle remain in the Faro division. The Kapsiki, another taurine cattle breed, with a higher introgression of Zebu genes, found mainly in the Mayo Tsanaga (Rhumsiki) area of the Far North region and also being on the verge of becoming extinct, were earlier shown to be trypanosusceptible [26].

The present research used both microscopy and molecular methods to study the occurrence and genetic diversity of trypanosomes in cattle from two agro-ecological zones (AEZ), focusing on areas with and without tsetse vectors [28].

## Results

### Body condition and packed cell volume in relation to breed and age

**Fig. 1:**
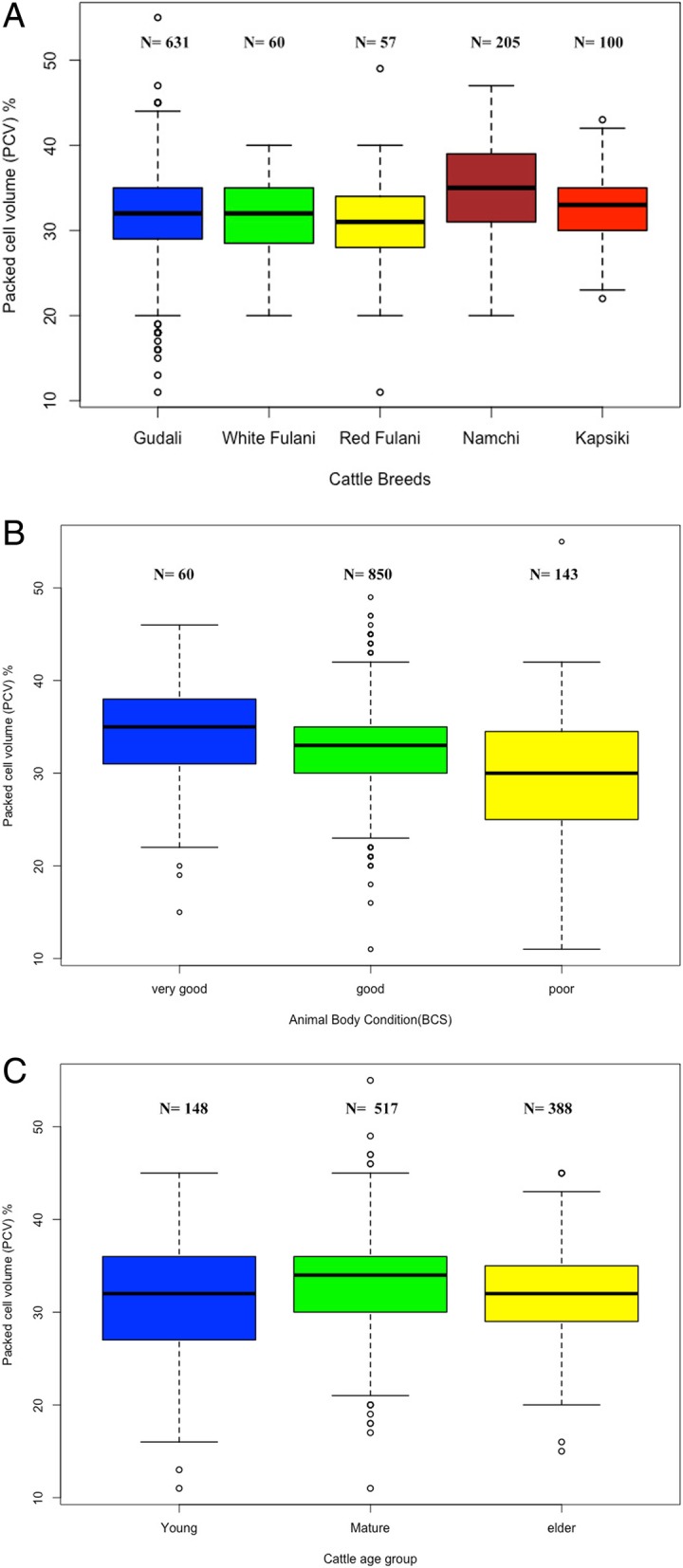
Effect of cattle breed on packed cell volume (**a**). Comparison of the mean of PCV of five indigenous cattle breeds examined. Effect of body condition score on packed cell volume (**b**). Animals were grouped as described in the section “Materials and Methods” without breed distinction and the PCVs were compared. Effect of age group on body condition score (**c**). Animal were grouped by age as described in in the section “Materials and Methods” and PCV was compared. Details of sample collections and processing are indicated in the section “Materials and Methods”

A total of 1176 animals were randomly sampled. These comprised more female (907; 77.1%) than male (269; 22.9%). Examined animals were from five divisions as follows: Vina (*n* = 283), Faro et Deo (*n* = 196), Mayo Rey (*n* = 316), Faro (*n* = 176) and Mayo-Tsanaga (*n* = 205). In the Faro and Mayo-Tsanaga divisions, only the indigenous taurine breeds, Namchi (Doayo) and Kapsiki, respectively, were examined. Here, the mean PCV of Namchi (Doayo) was significantly higher (F = 13.88; *P* < 0.001) than that of Kapsiki (Fig. 1a, Additional file 3: Table S1). Overall, animals with poor body condition had PCVs (average 29.66 ± 6.68) significantly lower (F = 22.062, *P* < 0.001) than that of animals in good (32.82 ± 4.99) and very good (34.26 ± 5.46) condition (Fig. 1b). Young cattle aged between 0 to 2.5 years had significantly lower PCVs (31.22 ± 6.82) than the other age groups (F = 5.38, *P* = 0.005, Fig. 1c, Additional file 3: Table S2). 97.6% of the Kapsiki cattle had the best body condition score (4 or higher, Additional file 3: Table S1) as compared to those of other cattle breeds. Comparing the different regions, animals in the Faro et Deo division had a mean PCV (28,13 ± 5.76) that was significantly lower (F = 49.13, *P* < 0.001) than those found in the Faro division (34.74 ± 5.35; Additional file 3: Table S2).

### Parasitological and molecular detection of trypanosomes

Microscopic detection of motile trypanosomes showed that 56 blood samples (5.9% of 971 cattle) carried at least one trypanosome species (Table 1). The highest prevalence was recorded in Faro et Deo (15.8%), followed by Faro (4.5%), Mayo- Rey (3.5%) and Vina (2.3%). In the Mayo-Tsanaga region no microscopy was carried out due to insecure work environment.

**Table 1:** Distribution of trypanosome species detected by microscopy in the study area

		Trypanosome species									
Sites	N	Negatives	*Tb*	*Tb*-like	*Tv*	*Tc*	*Tc* + *Tv*	*Tc* + *Tb*	*Tv* + *Tb*	*Tc* + *Tb + Tv*	*T. spp.* prevalence (%)
Vina	265	259	4	2	0	0	0	0	0	0	2.3
Faro et Deo	196	165	7	0	2	4	5	7	1	5	15.8
Mayo-Rey	316	305	1	0	5	2	1	1	0	1	3.5
Faro	176	168	1	2	2	0	0	3	0	0	4.5
Total	953	897	13	4	9	6	6	11	1	6	5.9

*Tb*: *T. brucei*, *Tb*-like: *T. brucei*-like, *Tc*: *T. congolense*, *Tv*: *T. vivax.* Animals from Mayo Tsanaga area were not considered because microscopy data collection was not carried out at this location and one positive animal suspected to be hybrid was not included in this table

The most frequently identified trypanosome species was *T. brucei* spp., followed by *T. vivax* and *T. congolense* (Table 1). However, 7.1% of trypanosomes were not clearly identified according to their motility and morphological characteristics and were recorded as *T. brucei*-like trypanosome species.

In contrast, out of 1176 samples examined by ITS-1 nested PCR, 626 samples showed the presence of one or more trypanosome species, giving an overall prevalence of 53.2% (Table 2). The highest prevalence was recorded in Mayo-Tsanaga (67.8%), followed by Faro et Deo (59.2%) and lowest in Faro (34.1%). From the 56 samples classed positive by microscopy, 41 (71.9%) were also detected by nested PCR (Table 3).

**Table 2:** Distribution of trypanosome species detected by ITS-1 PCR in the study areas

		Trypanosome species												
Sites	N	Negatives	*Tb*	*Tv*	*Tc*	*Tth*	*Tc + Tv*	*Tc* + *Tb*	*Tb + Tv*	*Tc* + *Tb* + *Tv*	*Tc* + *Tb* + *Tth*	*Tc* + *Tth* + *Tv*	*Tc* + *Tb* + *Tth* + *Tv*	prevalence (%)
Vina	283	131	8	5	0	130	0	0	5	0	0	4	0	53.7
Faro et Deo	196	80	4	29	6	28	2	2	32	3	0	7	3	59.2
Mayo-Rey	316	157	36	39	6	28	2	0	42	4	0	1	1	50.3
Faro	176	116	8	4	1	33	1	1	8	0	1	3	0	34.1
Mayo Tsanaga	205	66	7	1	4	115	0	0	4	0	0	8	0	67.8
Total	1176	550	63	78	17	334	5	3	91	7	1	23	4	53.2

*Tb*: *T. brucei*, *Tc*: *T. congolense*, *Tth*: *T. theileri / T. grayi*, *Tv*: *T. vivax*

**Table 3:** Comparison of the diagnostic test results obtained by parasite microscopy and molecular (ITS-1 PCR) methods

	PCR									Total	*T. spp.*
	*Tb*	*Tv*	*Tc*	*Tth*	*Tc* + *Tv*	*Tb* + *Tv*	*Tth* + *Tv*	*Tc* + *Tb + Tv*	Negatives		overlap (%)
*Tb*	1	1	0	6	0	0	1	0	7	16	43.8
*Tv*	0	3	0	3	0	2	0	0	1	9	88.9
*Tc*	0	0	2	1	0	0	0	0	3	6	50.0
*Tc* + *Tv*	1	1	1	0	0	0	3	0	0	6	100.0
*Tc* + *Tb*	1	3	1	2	0	0	1	0	3	11	72.7
*Tc* + *Tb* + *Tv*	0	1	1	1	0	0	2	0	1	6	83.3
*Tv* + *Tb*	0	0	0	0	0	1	0	0	0	1	100.0
Negatives	47	72	8	206	3	14	64	3	468	885	47.1
Total	50	81	13	219	3	17	71	3	483	940	48.6

*Tb*: *T. brucei*, *Tc*: *T. congolense*, *Tth*: *T. theileri / T. grayi*, *Tv*: *T. vivax. T. spp: T. all species* Animals from Mayo Tsanaga region were not considered because microscopy was not carried out at this location. Only the animals with parasitological and molecular data were considered

### ITS-1 sequences analysis

Samples were identified according to ITS-1 amplicon size as described previously [19, 24] (Table 4). Three representative samples with a product size of 426 bp considered to be *T. brucei* spp. were sequenced and the results aligned to sequences retrieved from databases searches. The results showed that all sequences belonged to *Trypanozoon*, either to *T. brucei* spp. or *T. evansi.* They differ only in their maxi-circles DNA and additional species-specific markers are needed to distinguish these species. Additionally, six PCR amplicons in the range of 645 bp and considered to be *T. congolense* savannah or forest types were sequenced and showed a similarity of 73 to 85% with *T. congolense* isolates from South Africa and Gabon, respectively [GenBank: KX870079, KX452163]*.*

**Table 4:** Trypanosome ITS-1 amplicon sizes of different *Trypanosoma* spp.

*Trypanosoma* species	Amplicon size (bp)
*T. congolense savannah* ^a^	640
***T. congolense forest*** ^a^	**640**
*T. congolense kilifi*	562
***T. brucei brucei*** ^a^	**426**
*T. brucei rhodesiense*	426
*T. brucei gambiense*	426
***T. evansi*** ^a^	**426**
***T. vivax*** ^a^	**180 and 250**
***T. theileri*** ^a^	**320**
***T. grayi*** ^b^	**318**

The bold lettered species were found in this study

^a^source: Adams et al. [19]

^b^Ngomtcho et al. [24]

Interestingly, the PCR products of 180 bp and 250 bp (*n* = 6) both corresponded to *T. vivax* sequences isolated from Ethiopia ([GenBank: KM391818, KM391825], 91 to 93% identical). For PCR products in the range of 320 bp, out of 30 samples analyzed, 26 (87%) corresponded to *T. theileri* sequences published in Genbank (98 to 100% identical). The other four sequences (13%) matched with entries of *T. grayi* (90 to 96% identical) with closest similarity to *T. grayi* ANR4 isolated from a tsetse in The Gambia [TriTrypDB: JMRU01000589] and 94 to 99% identical with sequences [NCBI Blastn: MG255201, MG255205] obtained from cattle and tsetse in North Cameroon in Gamba and Kontcha, respectively [24].

### Genetic diversity of trypanosome species

In total, five different trypanosomes were identified: *T. congolense, T. brucei* spp., *T. theileri, T. grayi* and *T. vivax*, respectively, using ITS-1 makers and sequencing analysis (Table 4). Due to the inability to discriminate between *T. theileri* and *T. grayi* just on the basis of the ITS1 amplicon size, samples with amplicons in the range of 320 bp were considered as *T. theileri/T. grayi*. Molecular analysis showed these to be the most prevalent species in all five study areas (30.8%, *n* = 362/1176). *T. theileri/T. grayi* was also the species most often missed or misidentified for *T. brucei* or *T. congolense* by microscopic observation, followed by *T. vivax* (Tables 1, 2 and 3). The overall prevalence of mixed infections was 11.4% (*n* = 134/1176). Co-infections of *T. brucei* spp. and *T. vivax* were the most common (*n* = 91/1176), followed by triple infections with *T. congolense*, *T. vivax* and *T. theileri/T. grayi* (*n* = 23/1176). We found eight animals co-infected by *T. brucei* spp., *T. vivax* and *T. congolense* savannah/forest-type, and four animals co-infected by *T. brucei* spp.*, T. vivax, T. congolense* savannah/forest and *T. theileri/T. grayi* (Table 2).

### The effect of study site, breed and age on the prevalence of trypanosomosis and correlation with the body condition score

Doayo (Namchi) cattle from Faro were significantly less infected (34.6%; X^2^ = 51.78, *p* < 0.000) with any trypanosome species than the other taurine cattle Kapsiki (67.8%) and the Zebu breeds Gudali (54.1%), Red Fulani (58.1%) and White Fulani (54.1%). There was also a significant difference between the five sampled study sites. The overall trypanosome infection rate was higher in Mayo-Tsanaga (67.8%) than in Faro et Deo (59.2%). However, 56.2% of the infected animals in Mayo-Tsanaga were infected with *T. theileri/T. grayi*, compared to only 7.5% in Faro et Deo. In contrast, when looking only at the species classically considered to be pathogenic such as *T. congolense*, *T. brucei* spp. and *T. vivax,* these were most prevalent in Faro et Deo (44.9%), followed by Mayo-Rey (42.7%), Faro (15.3%) and Mayo-Tsanaga (11.7%). The area with lowest prevalence was Vina (7.8%) (Table 5), a former tsetse-cleared area.

**Table 5:** Effect of age, breed, study areas and body condition score on trypanosome prevalence

Factors	Prevalence by PCR		*X* ^2^	*P*-value	Prevalence by PCR	*X* ^2^	*P*-value
	N	overall (%)			Pathogenic (Tc + Tv + Tb) (%)		
Age							
Young (0–2.5)	171	92 (53.8)			54 (31.6)		
Mature (3–5)	574	332 (57.8)	11.93	0.003	159 (27.7)*	13.68	0.001
Old (6–12)	431	202 (46.9)*			83 (19.3)*		
Body condition							
Poor (0–2)	148	80 (54.1)			52 (35.1)		
Good (3–4)	967	516 (53.4)	0.449	0.799	220 (22.8)*	17.31	0.000
Very good (5)	61	30 (49.2)			24 (39.3)		
PCV							
PCV < 25	109	64 (58.7)	1.931	0.165	49 (45.0)	18.476	0.000
PCV > 25	944	488 (51.7)			241 (25.5)*		
Sex							
Male	283	147 (22.4)			84 (29.7)	5.76	0.018
Female	968	510 (77.6)	0.048	0.439	220 (22.7)*		
Breed							
Gudali	649	351 (54.1)			185 (28.5)*		
White Fulani	60	35 (58.3)			30 (50.0)		
Red Fulani	57	30 (52.6)	46.79	0.000	23 (40.4)*	58.19	0.000
Namchi (Doayo)	205	71 (34.6)*			34 (16.6)*		
Kapsiki	205	139 (67.8)*			24 (11.7)*		
Areas							
Vina	283	152 (53.7)*			22 (7.8)*		
Faro et Deo	196	116 (59.2)			88 (44.9)		
Mayo Rey	316	159 (50.3)*	47.28	0.000	135 (42.7)	166.41	0.000
Faro	176	60 (34.1)*			27 (15.3)*		
Mayo Tsanaga	205	139 (67.8)			24 (11.7)*		

Symbols: (*) indicates difference between variables

### Comparison of areas with or without *Glossina-*vectors

The overall prevalence of trypanosomes was similar or even higher in the tsetse free areas (Vina 53.7% and Mayo Tsanaga 67.8%, Tables 2 and 5) than in the *Glossina*-infested zones (Mayo-Rey 50.3%, Faro et Deo 59.2% and Faro 34.1%). *T. theileri/T. grayi* were the most abundant trypanosome species in the tsetse-free zones. In contrast, in the *Glossina*–infested areas *T. vivax*, *T. brucei* and *T. congolense* were the predominant species (Table 2).

Some *T. congolense, T. brucei and T. vivax* cases were even detected in the areas of Vina and Mayo-Tsanaga, although these areas have been declared tsetse-free (Table 2).

### Phylogenetic analysis of gGAPDH

**Fig. 2:**
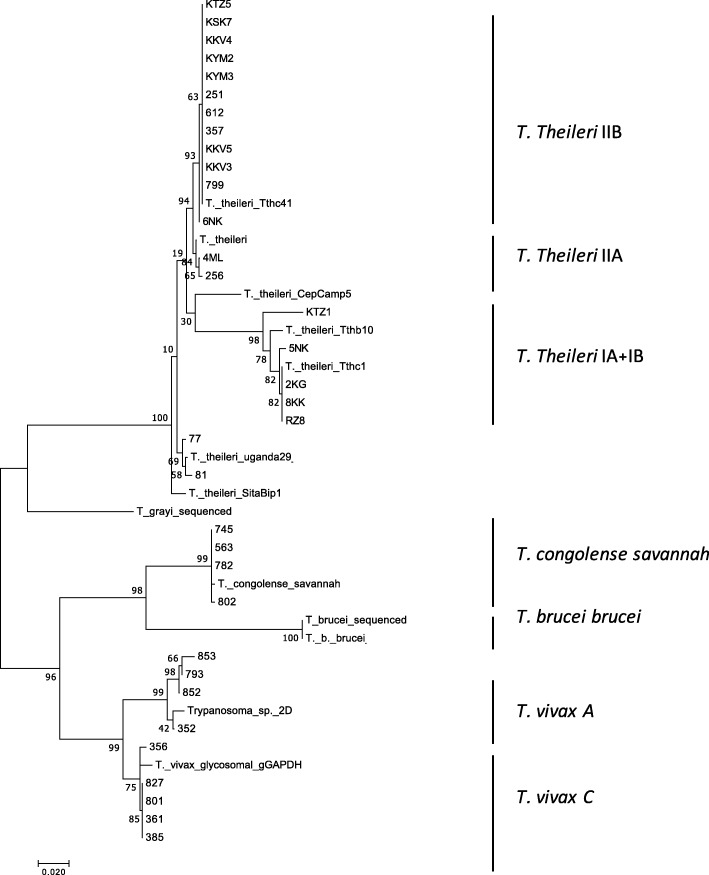
Molecular phylogenetic analysis by Maximum Likelihood method based on the gGAPDH–encoding gene sequence as detailed under “Material and Methods”. It contains an alignment of 535 bp stretches of 37 sequences obtained in this study plus reference sequences [HQ664796; FM164792; HQ664805; HQ664784, HQ664792; HF545654; FM164789; XM_840453; FN400713] retrieved from Garcia et al. [29] and Hamilton et al. [23]. The bootstrap support values (> 70% in 1000 replications) are shown for the nodes

Two main clusters were observed in the 37 gGAPDH sequences examined, comprising the stercorarian *T. grayi* and *T. theileri* on the one hand, and the salivarian *T. congolense*, *T. brucei brucei* and *T. vivax* on the other (Fig. 2). Interestingly, two clades of *T. theileri* were observed (IIB and IA/IB) as previously described [29]. Furthermore, the occurrence of two lineages was also observed in the main group of *T. vivax*, cluster C and cluster A [30]. Cluster C had previously been reported in various regions in Africa and America, while cluster A was described only in Tanzania [FM164789; FM164787]. *T. vivax* C and A were found co-infecting cattle in the Faro et Deo region.

### Correlation of packed cell volume with infection status

**Fig. 3:**
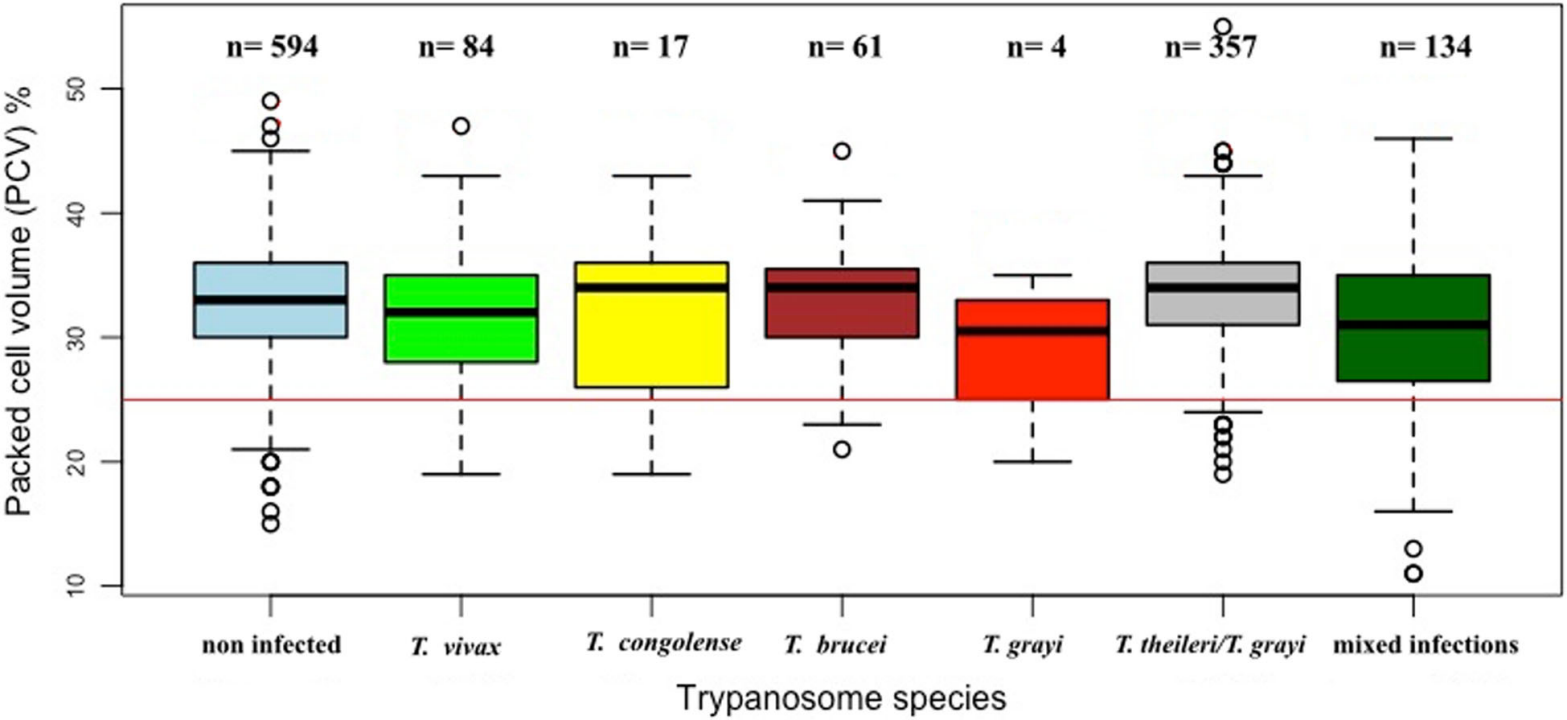
Effect of the species of trypanosomes detected by PCR on the Packed Cell Volume (PCV). Mixed infection is defined as the combination of at least two trypanosome species identified in the same animal. Details of sample collections and processing are indicated in the section “Materials and Methods”

Animals with single or mixed infections had lower PCV values when compared to those with no infection (Fig. 3). When comparing the mean PCV with the type of infection, animals with single-infections of *T. vivax* (31.68 ± 5.40) and *T. congolense* (31.29 ± 6.92) showed no significant differences from uninfected. Animals carrying *T. theileri* had a mean PCV of 31.9 ± 4.5 (*n* = 16) for clade IIB while that for clade IA and IB it was 35.8 ± 3.4 (*n* = 8) (Additional file 3: Table S3). The observed difference was close to significance (F = 2.043, *p* = 0.056). Animal infected with *T. grayi* had the lowest PCV (29 ± 5.5, *Ν* = 4) of all the groups. However, because of the small sample size of the *T. grayi* group we could not test for statistical significance.

## Discussion

The present study was carried out to determine the prevailing species and genetic diversity of trypanosomes infecting cattle in five divisions located in two agro-ecological zones of northern Cameroon, using both microscopy and molecular methods. The overall prevalence using microscopy is in agreement with previously reported prevalences of 3.7 to 20%, which were also determined by microscopy only [10]. However, infection rates determined by molecular analysis with ITS-1 nested PCR (53.2%) were much higher. This underpins the difficulty of microscopy to detect parasites at low levels of parasitemia in subclinical infections.

On the other hand, out of 56 trypanosome-positive cases by microscopy, only 41were detected by nested PCR giving the concordance rate of 73.2% between both techniques. This discrepancy has already been reported by Takeet et al. [31] and Adams et al. [19], the latter developing the primers used in our study. They also failed to amplify 56% of samples previously detected positive by microscopy and attributed this failure to the quality and quantity of the extracted parasite DNA. It is also possible that the primers do not amplify all trypanosome parasites [32, 33] or that *Borrelia* bacteria present in the blood are misinterpreted as trypanosomes, since based on their shape, size and movement, under the microscope they appear similar to *T. brucei* in buffy coat slide preparations [34]. Actually, recent molecular studies showed that 17.7% of cattle in the northern Cameroon are infected with *Borrelia theileri* (B. Abanda, A. Paguem, M. Abdoulmoumini, TK. Manchang, A. Renz and A. Eisenbarth. personal communications).

We distinguished only three species of trypanosomes by microscopy, namely *T. congolense, T. vivax* and *T. brucei* spp.*,* while others, which we named *T. brucei*-like, could not be identified beyond doubt. By using PCR, we were able to identify five species of trypanosomes in the study area. This can be explained by the high sensitivity of the generic primers (ITS-1), which can detect traces of DNA up to one parasite per mL of blood of both pathogenic and non-pathogenic species [17, 18]. In contrast, microscopy of the Buffy-coat extracted from a microcapillary tube can reliably detect motile parasites only at a concentration being higher than 1.25 × 10^3^ parasites/mL of blood [15–17]. Such high parasite titers in blood are more typical for trypanosomes causing pathology, like *T. brucei* spp., *T. congolense* and *T. vivax* at the acute clinical stage, and chronic infections are likely to be missed.

Surprisingly, the stercorarian parasites *T. theileri/T. grayi* were the most prevalent species (30.5%) in our study. These two parasites cannot be distinguished by ITS-1 size estimation, but only by sequence analysis, because they have a similar band size of 320 bp on the gel. Four out of 30 samples analyzed by sequencing were identified as *T. grayi* whereas the other 26 were *T. theileri*.

**Fig. 4:**
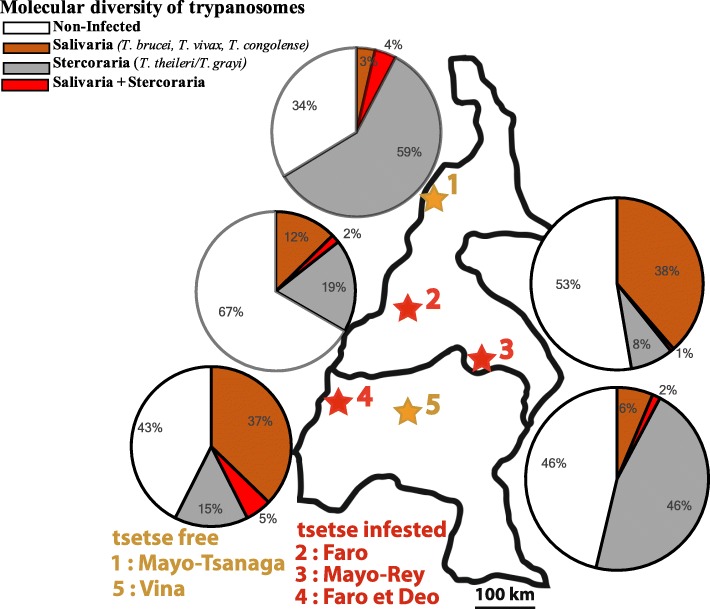
Distribution of Salivaria (*T. brucei, T. vivax* and *T. congolense*) and Stercoraria (*T. theileri/T. grayi*) in tsetse free and tsetse infested areas in Northern Cameroon. Details of sample collections and processing are indicated in the section “Materials and Methods”.(map depicted in Fig. 4 is from our own)

*Trypanosoma grayi* was found in two of 7 cattle from Mayo-Tsanaga and in one of 6 from Vina and yet another one from 12 cattle examined at Mayo-Rey. Previously, this species was known only to be a parasite of reptiles [1]. However, recently this parasite has been detected in a White Fulani cattle in Faro et Deo [24] and has now also been found in Kapsiki and Gudali cattle. By extrapolation on our 358 *T. theileri*/*T. grayi* cases we could expect almost 50 animals to be infected with *T. grayi*. This observation raises concerns whether these parasites may represent a strain undergoing a change of host range [24]. Further investigations are essential to characterize those *T. grayi* strains and evaluate their pathogenic potential for cattle and/or other livestock. In our study areas animals infected with this parasite correlated with lower PCV which may be an indicator of potential pathogenic effects on animal health. In this context, it is noteworthy that a recent study in Nigeria has observed a high frequency of tsetse colonised with *T. grayi*-like parasites (J. Weber. personal communication). Furthermore, these parasites revealed a high genetic diversity suggesting a dynamic evolution in this region. The 320 bp amplicon representative for the stercorarian parasites of *T. theileri/T. grayi* was most prevalent in the tsetse-free Vina (47.3%) and Mayo-Tsanaga (60.0%) regions and much less frequent in the tsetse-infested areas Faro (21.0%), Faro et Deo (19.4%) and Mayo-Rey (8.2%) (Fig. 4). This observation suggests that abundant mechanical vectors are the drivers of transmission of Stercoraria in the presumably tsetse-free areas [10, 35]. The entomological survey by Lendzele et al. [36] in the Vina and Mayo-Rey division identified seven species of tabanids as potential mechanical vectors: *Tabanus gratus*, *Ta. par*, *Ta. taeniola*, *Ta. biguttatus*, *Ta. sufis* and *Chrysops distinctipennis*. Furthermore, four prevailing species of tabanids were found in the Far North region: *Atylotus agrestis*, *Ta. taeniola*, *Ta. par* and *Ancala spec* [37].. Desquesnes and Dia [38, 39] have proved experimentally the mechanical transmission of *T. vivax* and *T. congolense* by tabanids (*Atylotus agrestis*). In addition, *Ta. par* and *Ta. taeniola* were tested PCR positive for the presence of *T. congolense*, *T. theileri, T. evansi* and *T. brucei* in South Africa and the Gambia [40]. Additionally, ixodid ticks were identified as vectors of *T. theileri* in Germany and in Sudan [4, 5]. However, to our knowledge no detailed studies on mechanical vectors have been performed in the study areas until now.

Infections with *T. brucei* spp. (5.0%) and *T. vivax* (6.7%) were the most prevalent classical pathogenic trypanosomes found in our study areas. They were significantly more prevalent in Faro et Deo and Mayo-Rey (Tables 1 and 2) compared to the other locations. This observation was expected, because Faro et Deo is situated between the tsetse-infested Gashaka Forest Reserve on the Nigerian border and the Faro Game Reserve, and Mayo-Rey between the hunting zones and the Bouba Ndjida National Park, which both harbor a large population of known reservoir species for trypanosomes (antelopes, buffalos, etc.) with particularly abundant tsetse populations [9, 41]. The high infection rate observed in Faro et Deo is in agreement with prevalences of 10 to 41%, obtained in earlier studies [7, 9, 10, 24]. In this area *Glossina morsitans submorsitans* and *G. palpalis palpalis* are the main prevailing vector species [24, 41]. In Mayo-Rey, *G. tachinoides* was also abundant, together with *G. m. submorsitans* [9].

Lower prevalences of *T. vivax*, *T. congolense* and *T. brucei* spp. were observed in Mayo-Tsanaga (11.7%) and Vina (7.8%), both considered tsetse free. However, the presence of these trypanosomes may indicate presence of tsetse in these areas, perhaps due to re-infestation of tsetse from the nearby wildlife reserves which had not been subject to tsetse control. It can be as well due to the introduction of infected animals from tsetse infested zones.

*Trypanosoma congolense* was detected in the Vina (*n* = 4/283; 1.4%) and in Mayo-Tsanaga (*n* = 12/205; 5.8%) only by molecular methods, a status which does not exclude the activities of tsetse in these areas. In the Adamawa plateau prevalence of 3% by microscopy and 21% by serological tests were previously reported [42]. For the Mayo-Tsanaga division this is the first report of *T. congolense* in cattle. However, since no molecular confirmation was done before, it is possible that these infections were misidentified previously. Or they may have been recently introduced by infected tsetse or infected Fulani animals coming from transhumance through tsetse infested areas of neighboring countries like Nigeria.

*Trypanosoma vivax* sequence analysis revealed the occurrence of two phylogenetically distinct strains: *T. vivax* type C [30], previously described to be distributed across Africa and America, and *T. vivax* type A, which was isolated so far only in Tanzania [FM164789; FM164787]. In our study areas, we found both strains sympatric with other trypanosomes in the Faro et Deo division. The type A has been reported to be responsible for several outbreaks of bovine trypanosomosis in East Africa [30]. This raises the concern for potential outbreaks in the Faro et Deo region, and the potential to spread further throughout the country.

When looking at the PCV values, animals carrying mixed infections had significantly lower values than the non-infected and single-species infected animals. Furthermore, when comparing the sampling areas, Faro et Deo had the lowest PCV values both in infected and uninfected cattle. It has also to be considered that anaemia may be the result of other hemoprotozoan and/or helminths infections [43]. Infected Kapsiki cattle showed the lowest PCVs when compared to the other indigenous *Bos taurus* breed Doayo (Namchi). It has been previously reported [44] that the Doayo cattle were trypanotolerant while the Kapsiki were trypano-susceptible and this was associated with higher introgression of zebu alleles in the Kapsiki [26]. In a previous study, it was observed that *T. theileri* clade IIB, though considered non-pathogenic in cattle, correlated with low PCV in infected animals [24]. This tendency to become pathogenic was attributed to the genetic association to a previously described clade [29]. Comparing the PCV values of all animals in this study in which DNA of *T. theileri* was found, the mean PCV of cattle infected with clade IIB (31.9 ± 4.5) was slightly lower than those of animals with clade IA and IB (35.8 ± 3.4, *p* < 0.057). This implies that infections with clade IIB may be pathogenic to cattle and should be further investigated and considered during clinical control operations for cattle kept under local husbandry conditions. Once more, this underlines the importance to further investigate the development and evolution of trypanosome species, especially as these two clades of *T. theileri* are found worldwide. The prevalence of *T. brucei spp*, *T. vivax* and *T. congolense* in the tsetse-free areas of Mayo-Tsanaga and Vina raised questions whether the areas are still free and if tsetse control is sufficient enough to eradicate bovine trypanosomosis. Therefore, an entomological survey is urgently needed to check whether these previously tsetse-free areas have been re-infested by *Glossina* or whether these parasites are transmitted by non-tsetse vectors. Both scenarios call for close monitoring of the situation including molecular tools as used in this study as well as a thorough search for alternate vectors.

## Conclusions

Bovine trypanosomosis is more prevalent in the two ecological zones of northern Cameroon than previously thought. Five trypanosome species and subtypes were identified. Unexpectedly several cases of *T. grayi* were detected in cattle. Therefore, it may not be excluded that this parasite is already adapted to the cattle host. *Trypanosoma vivax*, clade A, which had previously only been identified in Tanzania was found to be co-endemic with *T. vivax* clade A and *T. vivax* clade C in the Faro et Deo region. Furthermore, the presence of two strains of *T. theileri,* clades IIB and IA/IB, was confirmed. This high diversity of *Trypanosoma* species makes monitoring and local control more complex than previously thought. Finally, the abundance of pathogenic trypanosomes in tsetse infested areas is alarming and even more, the occurrence of *T. vivax*, *T. brucei*, *T. congolense*, *T. theileri* and *T. grayi* in tsetse-free areas implies that tsetse control alone is not sufficient to control trypanosomosis in livestock.

## Methods

### Study areas

**Fig. 5:**
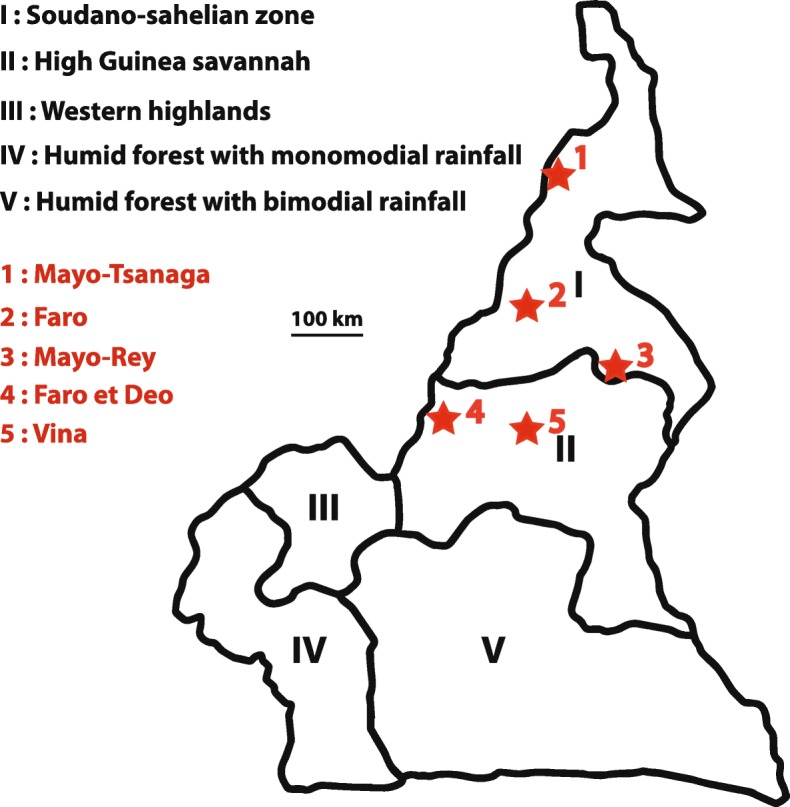
Map of the study area. Geographic map showing five Agro-Ecological Zones of Cameroon (based on information from the Institute of Agricultural Research for Development, IRAD, 2009). The cattle sampling areas (red stars) were located in the climate zones Guinea wet savannah and Sudano-Sahelian dry savannah. (map depicted in Fig. 5 is from our own)

This study was carried out in the Far North, North and Adamawa region of Cameroon (Fig. 5: Additional file 3: Table S4). These three regions are localized in two large Agro-Ecological Zones: the Sudano-Sahelian (Far North region and a larger part of the North region) and the Guinea savannah of the Adamawa plateau (Adamawa region with a little part of the North region). Cattle rearing is most abundant in the Guinea savanna of the Adamawa plateau with suitable climate and pasturelands for extensive cattle rearing. Overall, this plateau contributes to about 38% of beef production in the country [45]. The sampling sites were located in five divisions lying between latitudes 7 to 10°N and 11 to 15°E and covered an area of 164,000 km^2^ [46]. A strong climatic gradient runs through the wet high Guinea savannah in the Adamawa up to the dry Sudano-Sahelian zone in the Far North region. The rainy season in the Guinea savannah zone is from April to October, whereas in the Sudano-Sahelian zone it is from June to September. Annual rainfall ranges from 1400 to 1700 mm in the Guinean savannah and 800–1400 mm in the Sudano-Sahelian zone (Fig. 5).

### Experimental design and animal selection

A cross-sectional survey was carried out between April 2014 and June 2015. For each herd visited, about 10% of the animals were sampled using a systematic random method described by Dohoo et al. [47]. In the Faro and Mayo Tsanaga divisions only the indigenous taurine cattle breeds Doayo (Namchi) and Kapsiki, respectively, were examined and sampled. From each animal, physical examinations were made and the following variables recorded: breed, sex, body condition score (BCS) using the method described by Pullan for White Fulani [32], on a scale from 0 to 5 (0–2: poor condition, 3–4: good condition and 5 very good condition or fat), and age by dentition categorized as young (< 2.5 years), mature (> 2.5–5 years) and older (> 5 years). In many farms only very few males were present in the herds causing the random selection to be applied on the animals found in the herd without balancing for sex proportions.

### Assessment of packed cell volume (PCV) and trypanosome detection

Approximatively 5 mL of blood were collected from the jugular vein of each animal, using a vacutainer tube containing potassium ethylenediaminetetraacetic acid (EDTA) anticoagulant (VACUETTE® K3 EDTA). The samples were stored in a cooler box until processing within 6 h after collection either at a stationary or mobile laboratory in the field. Plasma was separated from blood by centrifugation at 3000 rpm for 15 min. Then the buffy coat was carefully collected and stored at 4 °C for subsequent DNA extraction. To determine the PCV, blood was introduced into capillary tubes (approx. 70 μL), and after sealing one end of the capillary tube with cristoseal (Sigma Aldrich, Germany) it was centrifuged at 12,000 rpm for 5 min using a microhaematocrit centrifuge (Hawksley, UK). The PCV was measured with a haematocrit reader (Hawksley Limited, UK). Animals that had a PCV value equal or less than 25% were considered anaemic. Subsequently, the capillary tube was cut with a diamond cutter 0.5 mm below the buffy coat to transfer the layer of white blood cells containing accumulated haemoparasites [16, 17] on to a clean microscope slide. After applying a coverslip over the buffy coat, approximately 200 fields of the preparation were examined for the presence of motile trypanosomes with a compound light microscope using 400x magnification [15]. The trypanosome species were classified according to previously described morphological criteria [14].

### Genomic DNA extraction, purification, PCR amplification, sequencing of ITS-1 and gGAPDH

Genomic DNA from buffy coat was extracted using the Wizard Genomic DNA Purification Kit (Promega, Germany) according to the manufacturer’s instructions, and then stored at.

− 20 °C. Generic primers were used in a nested PCR targeting kinetoplastid ITS-1 as described previously [19, 24]. Briefly, the first reaction (25 μL final volume) contained 2 μM of each outer primers (Table 6), 0.2 mM dNTP mix, 0.5 U Dream *Taq* DNA polymerase (Thermo Scientific, Dreieich, Germany), 1× Dream *Taq* buffer, and 1 μL of extracted DNA. Nuclease-free water and genomic DNA of *T. brucei*, *T. congolense* or *T. grayi* were used as negative and positive controls, respectively. PCR amplification was carried out as follows: initial denaturation step at 95 °C for 60 s, followed by 30 amplification cycles at 94 °C for 60 s, at 52 °C for 60 s, at 72 °C for 30 s, and final extension at 72 °C for 5 min. Thereafter, the second PCR reaction was carried out with 1 μL of first PCR product diluted 80-fold as template under the same cycling conditions as described above, except for an annealing temperature of 54 °C, and using the inner primer pairs (Table 6). 20 μL of the resulting PCR product was loaded onto a 2% TBE agarose gel stained with 0.5 μg/mL of DNA Stain G (SERVA, Heidelberg, Germany). Positive PCR amplicons of variable fragment sizes representing different trypanosome species (Table 4, Additional file 1: Figure S1) were randomly selected for Sanger sequencing. For these samples, the second reaction was carried out in a total volume of 50 μL with 2 μL of 80-fold diluted first PCR product.

**Table 6:** Generic Primers used for PCR amplification

Primers	5′- 3′ sequence	Sequence length (bp)
ITS1-OutF^a^	CTTTGCTGCGTTCTT	660–180
ITS1-OutR^a^	TGCAATTATTGGTCGCGC	
ITS1-InF^a^	TAGAGGAAGCAAAAG	
ITS1-InR^a^	AAGCCAAGTCATCCATCG	
gGAPDH-OutF^b^	TTYGCCGYATYGGYCGCATGG	900
gGAPDH-OutR^b^	ACMAGRTCCACCACRCGGTG	
gGAPDH-InF^b^	CGCGGATCCASGGYCTYMTCGGBAMKGAGAT	
gGAPDH-InR^b^	GTTYTGCAGSGTCGCCTTGG	

Primer. In: inner primer, Out: outer primer, F: forward, R: reverse,

^a^Adams et al. [19]

^b^Hamilton et al. [23]

An approximately 900 bp region of the gGAPDH gene was amplified by nested PCR and sequenced using the primers described by Hamilton et al. [23]. Nested PCR was carried out using 2x Red Mastermix (Genaxxon Bioscience, Ulm, Germany) to generate PCR products for direct sequencing. Briefly, the first PCR reaction with a final volume of 25 μL contained 1x mastermix, 0.5 μM of outer primers (Table 6), and 2 μL of genomic DNA template under the following conditions: initial denaturation at 95 °C for 3 min, 30 cycles at 95 °C for 1 min, annealing at 55 °C for 30 s, elongation at 72 °C for 1 min, followed by a final elongation step at 72 °C for 10 min. The first PCR products were diluted 80-fold and 2 μL transferred to the second PCR reaction with the inner primers (Table 6, Additional file 2: Figure S2) under the same conditions as the first reaction. Amplified products were subjected to electrophoresis on 2% agarose gels. The selected positive PCR products were sent for sequencing (Macrogen, Netherlands).

A subset of positive amplicons was excised from the gel and purified using GeneJet Gel Extraction Kit (Thermo Scientific, Dreieich, Germany) according to the manufacturer’s instructions. DNA concentrations were determined by photometry on a Nanodrop 1000 (Thermo Scientific, Dreieich, Germany) before submitting them to a commercial sequencing provider (Macrogen).

### Statistics and phylogenetic analysis

The results from the parasitological and molecular approaches were compared by Chi-Square tests to assess the association between prevalence, breed, BCS, sampled area and age group. Fisher’s Exact Test was done to compare mean PCV values. Since only 269 (23%) samples were collected from male, no sex-differentiating analysis was performed.

Differences were tested for significance at *p* < 0.05 using the statistical software program SPSS v.25.0.0 (IBM, USA). Obtained sequences were analyzed using Geneious (Biomatters, Auckland, New Zealand) and aligned to sequences retrieved from data bases searches (GenBank, NCBI, https://blast.ncbi.nlm.nih.gov/genbank/), and TriTrypDBv.6.0 (http://tritrypdb.org) using nucleotide BLAST.

To investigate the genetic diversity of trypanosomes present in the study area, and to analyze their phylogenetic relationship in order to detect subpopulations of trypanosomes restricted to respective study areas, gGAPDH was used as a marker locus. Phylogenetic trees were aligned and constructed by MEGA7 software [48], and the evolutionary history was inferred using the Maximum Likelihood method (ML) based on the Kimura 2-parameter model [49]. Confidence in branching relationships was assessed using bootstrap re-sampling over 1000 replicates. The final construct nucleotide length used in this analysis was 535 bp.

## Supplementary information

**Additional file 1: Figure S1.** PCR amplicons of *Trypanosoma* species in northern Cameroon. ITS amplicon sizes of different *Trypanosoma* species in the range of 200 to 650 bp. The amplicons were resolved on a 2% TBE agarose gel. The first lane shows the marker (M), the second lane shows the ITS-1 fragment for *T. vivax* (Tv) at 200 bp and a faint band at around 180 bp. The third and fourth lanes show the presence of two species, *T. theileri* and *T. grayi* (Tth/Tg) at 380 bp, the fifth line *T. brucei* spp. (Tb) at 400 bp and the sixth lane the presence of *T. congolense* forest type (Tcf) at 640 bp. C-1: Water control of 1st reaction, C-2: Water control of 2nd reaction.

**Additional file 2: Figure S2.** gGAPDH amplicons of different *Trypanosoma* species gave one band size of 900 bp. The first lane shows the marker (M), the second (852) and third (853) lanes are positives, the fourth (884), fifth (895), sixth (898) and seventh (849) lanes are negative samples. The eighth lane is the amplicon of *T. grayi* genomic DNA used as a positive control and C is double distilled water as a negative control.

**Additional file 3: Table S1.** Effect of cattle breed on animal body condition. **Table S2.** Effect of study area, cattle breed and age group on packed cell volume. **Table S3.** Packed cell volume of animals infected with *T. theileri* clade IA, IB and IIB. **Table S4**. GPS Coordinates of study areas and sampled herds.

## Data Availability

All data generated and analyzed during this study are included in this published article and its supplementary information files or available from the corresponding author on reasonable request. The sequences generated during the present study are available in the NCBI Genbank repository under the accession numbers MK674001-MK674048, MK656901-MK656904.

## Abbreviations

AEZ: Agro Ecological Zones
EDTA: Ethylenediaminetetraacetic acid
gGAPDH: glycosomal glyceraldehyde 3-phosphate dehydrogenase gene
ITS-1: Internal transcribed spacer 1 region of the ribosomal RNA gene locus
PCV: Pack cell volume
SPP: Subspecies
