# Supplementary figures and images for: Widespread co-endemicity of Trypanosoma species infecting cattle in the Sudano-Sahelian and Guinea Savannah zones of Cameroon
Source: BMC Vet Res. 2019 Oct 16;15:344. doi: 10.1186/s12917-019-2111-6 (PMC6796345; doi:10.1186/s12917-019-2111-6)

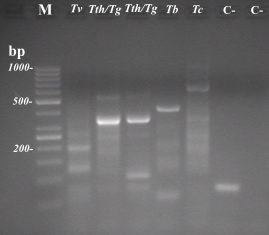

Supplement: Supplementary file 1 — Additional file 1: Figure S1. PCR amplicons of Trypanosoma species in northern Cameroon. ITS amplicon sizes of different Trypanosoma species in the range of 200 to 650 bp. The amplicons were resolved on a 2% TBE agarose gel. The first lane shows the marker (M), the second lane shows the ITS-1 fragment for T. vivax (Tv) at 200 bp and a faint band at around 180 bp. The third and fourth lanes show the presence of two species, T. theileri and T. grayi (Tth/Tg) at 380 bp, the fifth line T. brucei spp. (Tb) at 400 bp and the sixth lane the presence of T. congolense forest type (Tcf) at 640 bp. C-1: Water control of 1st reaction, C-2: Water control of 2nd reaction. [file 12917_2019_2111_MOESM1_ESM.tiff]

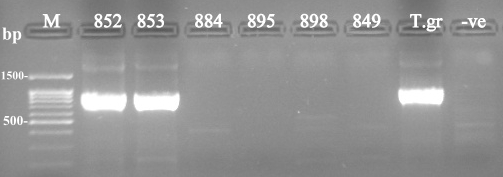

Supplement: Supplementary file 2 — Additional file 2: Figure S2. gGAPDH amplicons of different Trypanosoma species gave one band size of 900 bp. The first lane shows the marker (M), the second (852) and third (853) lanes are positives, the fourth (884), fifth (895), sixth (898) and seventh (849) lanes are negative samples. The eighth lane is the amplicon of T. grayi genomic DNA used as a positive control and C is double distilled water as a negative control. [file 12917_2019_2111_MOESM2_ESM.jpg]
